# Supplementary material for: Content-rich biological network constructed by mining PubMed abstracts
Source: BMC Bioinformatics. 2004 Oct 8;5:147. doi: 10.1186/1471-2105-5-147 (PMC528731; doi:10.1186/1471-2105-5-147)
Supplement: Additional File 2 — The original results of the above study (non-essential files are deleted to keep the file size under the limit set by BMC bioinformatics). [file 1471-2105-5-147-S2.bz2 › chilibotAdditionalFile2/dip30/138ID7739537E494/html/TFDP1_E2F2.html]

 


 **TFDP1** and **E2F2** 
  
Found 26 abstracts in PubMed, retrieved 26.  
 

 What does Google say? 
 PDF only 
| .edu only 

---

**Interactive relationship** (e.g. stimulation, inhibition, etc)

**Neutral relationship**- E2F 4 and 5 share common sequences with E2F 1, E2F 2  [ **E2F2** ] , and E2F 3 and, like these other E2Fs, the ability to heterodimerize with DP 1  [ **TFDP1** ] , thereby acquiring the ability to bind an E2F DNA recognition sequence with high affinity.  Ref: 7892279 Proc Natl Acad Sci U S A, 1995
- Both DP 1  [ **TFDP1** ]  and DP 2 synergistically interact with members of the E2F family of proteins, E2F 1, E2F 2  [ **E2F2** ] , and E2F 3, to generate DNA binding complexes that specifically recognize the E2F site and functionally interact with E2F 1 in E2F site dependent transcriptional activation of cellular genes.  Ref: 7865876 Mol Biol Cell, 1994

**Non-interactive relationship** (e.g. studied together, co-existance, homology, etc.)

- For both E2F 1 and E2F 2  [ **E2F2** ] , but not E2F 3 or E2F 5 DP1  [ **TFDP1** ] , cell cycle re entry was associated with almost quantitative cell death.  Ref: 9780003 Oncogene, 1998
- In transfected cells, DP 1  [ **TFDP1** ]  did not accumulate in the nucleus unless it was coexpressed with the heterodimeric partners E2F 1, E2F 2  [ **E2F2** ] , or E2F 3.  Ref: 8832394 J Cell Sci, 1996
- In extracts from log phase cells, E2F complexes contained predominantly E2F 4 and E2F 2  [ **E2F2** ]  in association with DP 1  [ **TFDP1** ] , and DNA binding assays showed complexes containing E2F 2  [ **E2F2** ]  preferentially interact with only one of the two overlapping E2F sites.  Ref: 9020173 J Biol Chem, 1997
- This region of E2F 1 is conserved in E2F 2  [ **E2F2** ]  and E2F 3, and deletion of this region drastically reduces the transcriptional activity of the molecule without affecting DP 1  [ **TFDP1** ]  binding, suggesting that this region of the E2F transcription factors is involved in regulating their activity.  Ref: 8035503 J Virol, 1994
- Severe deregulation of G1 S transition was also indicated by the dramatic up regulation, particularly in the HCCs, of pRb free E2F1 DP1  [ **TFDP1** ]  and  **E2F2**  DP1  [ **TFDP1** ]  transcription factor heterodimers, as assessed by immunoprecipitation and immunohistochemistry.  Ref: 9426068 Cancer Res, 1998
- No differences were observed between RA sensitive and RA resistant ovarian carcinoma cells in the levels of expression of many cell cycle genes including cyclin A, B and E, cdk and 6, E2F 1, E2F 2  [ **E2F2** ] , E2F 3, E2F 4, E2F 5, DP 1  [ **TFDP1** ]  and DP 2.  Ref: 11753676 Oncogene, 2001
